# Research and development investments for biologics independently developed by US biotechnology startups, 2017–2023

**DOI:** 10.1093/haschl/qxaf139

**Published:** 2025-07-25

**Authors:** Ilina C Odouard, So-Yeon Kang, Jessica Mo, Gerard F Anderson, G Caleb Alexander

**Affiliations:** Department of Health Policy and Management, Johns Hopkins Bloomberg School of Public Health, Baltimore, MD 21205, United States; Department of Health Management and Policy, School of Health, Georgetown University, Washington, DC 20007, United States; Johns Hopkins University School of Medicine, Baltimore, MD 21205, United States; Department of Health Policy and Management, Johns Hopkins Bloomberg School of Public Health, Baltimore, MD 21205, United States; Division of Internal Medicine, Johns Hopkins University School of Medicine, Baltimore, MD 21205, United States; Department of Epidemiology, Johns Hopkins Bloomberg School of Public Health, Baltimore, MD 21205, United States

**Keywords:** biotechnology, venture capital, innovation, research and development, investment

## Abstract

**Introduction:**

Despite policy interest in pharmaceutical innovation, little is known about the investment needed for venture capital–backed startups to develop innovative biologics, a growing segment of the pharmaceutical marketplace.

**Methods:**

In a cross-sectional analysis of Food and Drug Administration (FDA) drug approval data, investment deal records, and clinical trials data, we estimated the investment needed for a biotechnology startup company to independently develop a biologic approved by the US FDA with priority review, an indicator of innovation. To isolate a homogenous set of cases, the sample focused on independent drug development of 13 drugs by 9 biotechnology startup companies that retained ownership of the drug from the start of development to FDA approval without being acquired or licensing the product.

**Results:**

We found that the median investment per FDA-approved biologic was $304.1 million (IQR: $289.9–$790.3 million) in uncapitalized costs, accounting for the cost of failures.

**Conclusion:**

This estimate represents the direct drug R&D investment needed for independent development of innovative biologic products by biotechnology startup companies.

## Introduction

There is policy concern that lower drug prices resulting from the Inflation Reduction Act (IRA) negotiation program and Prescription Drug Affordability Boards will negatively affect pharmaceutical innovation. As a result, the US Congressional Budget Office (CBO) and the US House of Representatives Budget Committee have renewed interest in the financial resources needed for drug research and development (R&D).^1,2^

These concerns represent the latest iteration of decades-long policy interest in pharmaceutical innovation. Legislation has shaped the market for drug development in numerous ways: the Bayh-Dole Act (1980) allowed academic inventions to spin off into companies, the Small Business Innovation Research (SBIR) program created in 1982 provides grants to small businesses developing technological innovations, the Orphan Drug Act (1983) introduced incentives for the development of drugs treating rare diseases, and the Biologics Price Competition and Innovation Act (2009) encouraged innovation and competition among biologics and biosimilars.^3-6^

Shaped by these policies, the types of drugs developed have evolved. Although small molecules have historically been more common, biologics represent a growing proportion of new Food and Drug Administration (FDA) approvals, reaching nearly 50% in 2023.^7^ Biologics are protected by more patents than small molecules, have higher clinical and regulatory success rates, and have a median period of market exclusivity of 20.3 years compared with 12.6 years for small molecules.^8^ Orphan drug designations have also grown more than 4-fold from the 1990s to the 2010s.^9^

Companies have also developed new models of drug development. Drug R&D has increasingly shifted from in-house development by large companies towards development by smaller companies, including biotechnology startups.^10^ Biotechnology companies and universities account for half of innovative new drugs, and venture capital (VC) is one of the fastest growing sources of funding for biopharmaceutical R&D.^11,12^ Biotechnology startups without established revenue streams must cover all R&D costs by raising capital from investors such as VC firms during early-stage R&D. If the drug under development is promising, the startup may eventually be acquired by an established pharmaceutical company, which commercializes the drug, or become publicly traded and commercialize the drug independently or in partnership with an established pharmaceutical company.^12^

Most prior studies examining R&D financing have focused on drugs commercialized by publicly traded, established companies and used reported R&D expenditures and transaction prices from publicly available Securities and Exchange Commission (SEC) filings. These estimates cover a broad range of drug types and range between $757 million and $2.6 billion,^13-17^ accounting for the cost of capital, which represents the opportunity cost.^18^ Studies that focused on biologics examined cell and gene therapies and oncology drugs,^15,16^ while other larger studies did not restrict the sample to particular types of drugs.^13,14^

These studies represent both drugs developed in-house by large companies as well as drugs that changed ownership over the course of development from smaller private companies to larger public companies. Changes in ownership present a challenge in estimating the cost of R&D related to disentangling the proportion of acquisition prices and licensing fees attributable to R&D expenditures vs expected future returns or other commercial expenses. Prior studies used acquisition prices and licensing fees in the absence of direct information on R&D financing among private originator companies, but considered these figures lower-quality proxies for the cost of R&D compared with R&D expenditures reported in SEC filings.^13^ For drugs developed independently by private companies without ownership changes, there are no acquisition prices nor licensing fees and SEC filings are not available. Due to these data limitations, the cost of R&D among this group of drugs has not yet been estimated.

Based on the policy interest in incentivizing innovative drug development, the increasing relevance of biologics, and the lack of information on R&D among drugs developed and commercialized by biotechnology startups, further research is needed to understand the costs and time required for biotechnology startups to develop new innovative biologics. Although this represents a smaller group of drugs, this “startup” model of drug development remains poorly understood despite being a source of increasing investment. Examining these companies also presents an opportunity to estimate R&D costs without the challenge of extracting R&D expenditures from transaction fees associated with changing ownership.

Using a novel dataset sourced from a proprietary investment history database, drug approval data, and clinical trial records, we estimated the investment needed for a biotechnology startup with a history of VC funding to develop an FDA-approved innovative biologic. We use this estimate as a proxy for the direct capital needed for R&D from the perspective of investee companies.

## Data and methods

### Data

We conducted a cross-sectional study using 4 databases to gather information on innovative biologic drugs approved by the FDA between 2017 and 2023. First, we used FDA drug approval databases to identify biologic approvals with priority review.^19,20^ Second, for each company owning an innovative FDA-approved biologic during our window of interest, we used Cortellis Competitive Intelligence, a biopharma intelligence database containing information about biopharma companies, drug pipelines, and approved drugs, to determine company ownership of each drug over time.^21^ Third, we used PitchBook to confirm company startup status and obtain each company's investment deal history. PitchBook tracks all investment deals among companies with a history of VC backing over time across all industries and has been used in prior peer-reviewed research on VC activities.^22-24^ Deal types in the dataset include VC, grants, public offerings, corporate investment, and mergers and acquisitions. Finally, we used ClinicalTrials.gov and FDA drug labels and approval documents to obtain development milestones: clinical trial start dates, priority review dates, and FDA approval dates.^25^

### Product selection

We first identified all biologics with priority review approved by the FDA between January 2017 and December 2023 (Figure S1). Priority review is an expedited review pathway for products considered by the FDA to represent a significant improvement in safety or effectiveness compared with standard of care,^26^ which we used as a proxy for innovativeness. We used priority review for 2 reasons: first, priority review has been used as a measure of innovation in prior literature^11^; second, priority review is the most common regulatory incentive for biologic products (>60%) and many products with other less common regulatory designations (eg, breakthrough therapy) come to the market through priority review.^19,27-29^ Thus, this strategy allowed us to avoid excessively limiting our sample while still capturing a set of drugs that meet the innovation standard necessary for faster approval.

We then excluded drugs that were discontinued for lack of clinical benefit (eg, aducanumab [brand name: Aduhelm]) and vaccine (eg, COVID-19 vaccine [Spikevax]) or tissue (eg, allogeneic processed thymus tissue [Rethymic]) products. We used the resulting list of 91 “index biologics” to identify startups for inclusion in the sample.

### Startup selection

We focused on the independent drug development cases, which are defined as biotechnology startups that retained ownership of their drugs from the start of development to FDA approval without being acquired or licensing the product (“startup” model). While this criterion limits the sample, this approach both represented an understudied and increasingly important segment of the pharmaceutical innovation marketplace. This limited sample is the most homogeneous set of cases to estimate the capital investment needed to develop drugs among biotechnology startups. Including drugs that changed ownership would have introduced acquisition prices and licensing fees, which reflect not only R&D costs but also expected future returns.

We used 3 criteria to determine which biologics were developed by startups and had not changed ownership. First, using Cortellis data, we required companies to have been privately held at the start of development of the index biologic, where start of development was defined as the first recorded evidence of the index biologic's existence (drug discovery date, first reported preclinical results, or receipt of regulatory designation). Second, we required companies to have retained ownership of the index biologic from the start of development until FDA drug approval. In 3 cases (Spark Therapeutics, Ultragenyx, and Y-mAbs Therapeutics), we made an exception to this requirement, as the originator was recorded as an academic institution and the drug candidate was later transferred to the company during the period when the company was privately held. As a result, the first financing date occurred after the start of clinical trials for these 3 companies, which could have led to underestimation of the total cost of developing these companies’ drugs. Finally, we required manufacturers to be startups, with a record of VC funding in PitchBook data. This resulted in a sample of 13 drugs from 9 companies. Two of the companies—bluebird bio and Ultragenyx—developed more than 1 index biologic. One company, argenx, also developed 1 other biologic in addition to the index biologic, but the additional biologic did not qualify as an index biologic because it lacked priority review.

### Outcomes

We defined the period from each company's first financing transaction to FDA approval of their index biologic as our analysis period. Our primary outcome was the direct investment needed for R&D, measured as the dollar amount invested in biotechnology startup companies per FDA-approved biologic during the analysis period. This included all completed deals during the analysis period, which spanned various types, including seed funding, VC deals, grants, public offerings, and corporate investment. After converting the deal transaction amounts to 2023 dollars using the Consumer Price Index,^30^ we summed the total investment amount during the analysis period for each company. These companies did not have profitable revenue streams and were focused solely on developing new drugs. Therefore, investments are assumed to represent the direct cost of new drug R&D, from the investee startup's perspective.

We calculated the investment amount required to develop an FDA-approved biologic for each company by dividing the total investment amount by the number of FDA-approved biologics developed by the end of the analysis period. By dividing the total investment by the number of FDA-approved drugs, we account for the potential investment in failed candidates that did not reach FDA approval during the period. All FDA-approved products were included in this calculation regardless of whether they were index biologics because it was assumed that capital raised by the companies was used for R&D for both index and non-index drugs. A similar approach to capturing the cost of failure was used in a prior study, where all of a company's R&D outlays until the company's first FDA-approved drug represented the cost of development, including failures.^16^

We report the investment amount per drug in both uncapitalized and capitalized costs. Uncapitalized costs represent the startup company's perspective, as we assumed biotechnology startup companies have no opportunity cost—that is, no other investment opportunities aside from investing the capital they raise in their R&D activities. Capitalized costs represent the investor perspective to facilitate comparison with other studies. We used a cost of capital rate of 10.5% per year, consistent with prior studies.^13,14^

We also quantified the number of years between development milestones, the investment amount received in each phase of development, and the proportion of total capital raised during each interval. We used the development milestones of each company's index biologic to calculate these outcomes. For the 2 companies with more than 1 index biologic, we used the development milestone dates of the company's most recently approved and independently developed index biologic. Companies were excluded from the calculation wherever clinical milestone dates did not exist. For example, a company without any phase 2 trial would not be included in the calculation of median time between phase 1 and phase 2.

We extracted data using Microsoft Excel, version 16.85 (Microsoft Corporation), and analyzed data using R, version 4.4.1 (R Foundation for Statistical Computing).

## Results

### Biotechnology startup characteristics

We identified 9 biotechnology startups with at least 1 product fulfilling our inclusion and exclusion criteria (“index biologics”) (Table 1). Most of the companies had only 1 index biologic, whereas Ultragenyx had 2 and bluebird bio had 4. All 9 companies became publicly traded during the period from first investment to most recent index biologic FDA approval.

**Table 1.** Biotechnology startup companies with biologics approved by the US Food and Drug Administration with priority review, 2017–2023 (*n* = 9 companies).

Manufacturer	Focus area	Year founded	Year of initial public offering	No. of FDA-approved drugs	Current company status
argenx	Immunology	2008	2014	2	Neither profitable nor acquired
bluebird bio	Cell and gene therapies	1992	2013	4	Acquired by private equity firm for $29 million in 2025
Gamida Cell Ltd	Cell therapy, oncology	1998	2018	1	Filed for bankruptcy in May 2024
Krystal Biotech, Inc	Genetic diseases	2015	2017	1	Profitable as of 2023
Portola Pharmaceuticals	Hematology, oncology	2003	2013	1	Acquired by Alexion Pharmaceuticals for $1.4 billion in 2020
Spark Therapeutics	Gene therapy	2013	2015	1	Acquired by Roche for $4.3 billion in 2019
Stemline Therapeutics	Oncology	2003	2013	1	Acquired by Menarini Group for $677 million in 2020
Ultragenyx Pharmaceuticals	Ultra-rare diseases	2010	2014	2	Neither profitable nor acquired
Y-mAbs Therapeutics	Immuno-oncology	2015	2018	1	Neither profitable nor acquired

Sources: Company websites (focus area, founding year, number of FDA-approved drugs), PitchBook investment deal data (year of IPO), SEC filings (current company status).

Abbreviations: FDA, US Food and Drug Administration; IPO, initial public offering; SEC, Securities and Exchange Commission.

Thirteen FDA-approved index biologic drugs were developed by these 9 companies (Table 2). The drugs were indicated for serious medical conditions, including cancers such as multiple myeloma and neuroblastoma, genetic disorders such as sickle cell anemia and X-linked hyperphosphatemia, and immune disorders such as myasthenia gravis. In addition to priority review, the drugs all had orphan designation and at least 1 other regulatory designation: fast track (*n* = 6), breakthrough therapy (*n* = 9), rare pediatric disease (*n* = 8), regenerative medicine advanced therapy (*n* = 2), and accelerated approval (*n* = 2).

**Table 2.** Development milestones of index biologics approved by the US Food and Drug Administration with priority review, 2017–2023 (*n* = 13 products).

Manufacturer	Drug name	Indication	Date of clinical trial start^a^			Priority review granted	FDA approval	Other regulatory designations
			Phase 1	Phase 2	Phase 3			
argenx	Efgartigimod alfa and hyaluronidase (Vyvgart Hytrulo)	Myasthenia gravis	10/11/2017	12/30/2016	8/22/2018	11/22/2022	6/20/2023	Orphan drug, fast track
bluebird bio	Idecabtagene vicleucel (Abecma)	Multiple myeloma	12/22/2015	10/14/2016	4/16/2019	9/22/2020	3/26/2021	Orphan drug, breakthrough therapy
bluebird bio	Betibeglogene autotemcel (Zynteglo)	Beta thalassemia	8/2013	8/2013	8/8/2016	11/22/2021	8/17/2022	Fast track, orphan drug, breakthrough therapy, rare pediatric disease
bluebird bio	Elivaldogene autotemcel (Skysona)	Cerebral adrenoleukodystrophy (CALD)	N/A	8/21/2013	8/21/2013	12/17/2021	9/16/2022	Orphan drug, breakthrough therapy, rare pediatric disease
bluebird bio	Lovotibeglogene autotemcel (Lyfgenia)	Sickle cell anemia	6/7/2013	6/7/2013	2/14/2020	6/21/2023	12/8/2023	Orphan drug, fast track, regenerative medicine advanced therapy, rare pediatric disease
Gamida Cell Ltd	Omidubicel (Omisirge)	Umbilical cord blood transplantation (hematologic malignancies)	11/2010	11/2010	12/16/2016	8/1/2022	4/17/2023	Orphan drug, breakthrough therapy
Krystal Biotech, Inc	Beremagene geperpavec (Vyjuvek)	Dystrophic epidermolysis bullosa	5/6/2018	5/6/2018	8/17/2020	8/18/2022	5/19/2023	Orphan drug, fast track, regenerative medicine advanced therapy, rare pediatric disease
Portola Pharmaceuticals	Coagulation factor Xa (recombinant), inactivated (Andexxa)	Reversal of anticoagulation	N/A	12/2012	3/2014	2/17/2016	5/3/2018	Orphan drug, breakthrough therapy, accelerated approval
Spark Therapeutics	Voretigene neparvovec (Luxturna)	Retinal dystrophy	9/2007	11/2010	10/2012	7/20/2017	12/19/2017	Orphan drug, breakthrough therapy, rare pediatric disease
Stemline Therapeutics	Tagraxofusp (Elzonris)	Blastic plasmacytoid dendritic cell neoplasm	9/2014	9/2014	9/2014	8/13/2018	12/21/2018	Orphan drug, breakthrough therapy
Ultragenyx Pharmaceuticals	Vestronidase alfa (Mepsevii)	Mucopolysaccharidosis VII	11/2013	11/2013	12/2014	5/23/2017	11/15/2017	Orphan drug, fast track, rare pediatric disease
Ultragenyx Pharmaceuticals	Burosumab (Crysvita)	X-linked hyperphosphatemia	12/2008	4/2011	10/22/2015	10/10/2017	4/17/2018	Orphan drug, breakthrough therapy, fast track, rare pediatric disease
Y-mAbs Therapeutics	Naxitamab (Danyelza)	Neuroblastoma	5/27/2009	12/2012	N/A	6/2/2020	11/25/2020	Orphan drug, accelerated approval, breakthrough therapy, rare pediatric disease

Sources: Drug labels (indication), FDA summary review documents (CDER) and summary basis for regulatory action document (CBER) (priority review and FDA approval dates), ClinicalTrials.gov (clinical trial dates).

^a^Clinical trial dates represent the first clinical trial of each phase in the eventually approved indication. When dates for multiple phases are the same, this indicates combined phase trials (eg, phase 1/2 or phase 2/3).

Abbreviations: CBER, Center for Biologics Evaluation and Research; CDER, Center for Drug Evaluation and Research; FDA, Food and Drug Administration; N/A, no trial in that phase.

### Capital investment needed for new biologic approval

During the period from the first investment to the most recent index biologic approval, there was a median investment per FDA-approved biologic of $304.1 million (IQR: $289.9–$790.3 million) and the mean was $623.5 million (SD: $632.2 million) (Table 3). In capitalized costs, the median was $873.7 million (IQR: $435.4 million–$1.0259 billion) and the mean was $1.0382 billion (SD: $917.6 million). There was a wide range in the investment amount per FDA-approved biologic by company: Stemline Therapeutics had the lowest amount at $143.9 million while argenx had the highest amount at $2.2 billion. There were also differences between companies in the composition and timing of investments (Figure S2). Gamida Cell Ltd, Portola Pharmaceuticals, Spark Therapeutics, Ultragenyx Pharmaceuticals, and Y-mAbs Therapeutics had more seed and VC funding, whereas argenx, bluebird bio, Krystal Biotech, and Stemline Therapeutics relied primarily on public offerings and private investment.

**Table 3.** Investment amounts in biotechnology startups with biologics approved by the US Food and Drug Administration with priority review, 2017–2023 (*n* = 9 companies).

Manufacturer	First financing date	Last investment before index biologic approval	Index biologic approval date	Analysis period,^a^ y	Capital raised during period, $ millions	FDA-approved drugs, *n*	Investment per FDA-approved drug,^b^ $ millions	Capitalized investment per FDA-approved drug,^c^ $ millions
argenx	12/3/2009	3/28/2022	6/20/2023	13.5	$4331.7	2	$2165.8	$3038.3
bluebird bio	10/6/2004	9/8/2021	12/8/2023	19.1	$1797.5	4	$449.3	$873.8
Gamida Cell Ltd	5/1/2000	6/28/2019	4/17/2023	22.9	$289.9	1	$289.9	$874.5
Krystal Biotech, Inc	8/9/2017	5/19/2020	5/19/2023	5.7	$304.1	1	$304.1	$459.6
Portola Pharmaceuticals	10/30/2003	2/3/2017	5/3/2018	14.5	$908.7	1	$908.7	$2005.9
Spark Therapeutics	2013	6/15/2016	12/19/2017	4.9	$790.3	1	$790.3	$1026.0
Stemline Therapeutics	9/19/2003	5/16/2013	12/21/2018	15.2	$143.9	1	$143.9	$280.0
Ultragenyx Pharmaceuticals	6/20/2011	7/1/2016	4/17/2018	6.8	$594.8	2	$297.4	$435.5
Y-mAbs Therapeutics	1/8/2016	9/21/2018	11/25/2020	4.8	$262.5	1	$262.5	$350.6
All companies, median (IQR)	—	—	—	13.5 (5.7-15.2)	$594.8 ($289.9-$908.7)	1 (1-2)	$304.1 ($289.9-$790.3)	$873.7 ($435.4-$1025.9)
All companies, mean (SD)	—	—	—	11.9 (6.6)	$1047.0 ($1332.2)	1.5 (1.0)	$623.5 ($632.2)	$1038.2 ($917.6)

Sources: FDA drug approval databases (index biologic approval date), PitchBook investment deal data (investment amounts and deal dates), company websites (number of FDA-approved drugs).

^a^Duration between first financing to most recent index biologic approval.

^b^Total capital raised divided by number of FDA-approved drugs for each company.

^c^Investment amounts were capitalized at a cost of capital rate of 10.5% per year.

Abbreviation: FDA, Food and Drug Administration.

In a sensitivity analysis where we excluded the 3 companies that had acquired the biologics from academic institutions early in development, the median investment per FDA-approved biologic was $376.7 million (IQR: $293.4–$793.8 million) and the mean was $710.3 million (SD: $760.0 million). In capitalized costs, the median was $874.1 million (IQR: $563.1 million–$1.723 billion) and the mean was $1.2553 billion (SD: $1.0599 billion).

### Time needed for new biologic approval

The median (IQR) time between the manufacturer's first financing transaction and FDA approval among these drugs was 13.5 (IQR: 5.7–15.2) years (Table 4). The total time from the start of phase 1 trials to FDA approval was a median of 7.9 (IQR: 4.8–10.7) years. The start of phase 1 clinical trials occurred a median of 5.1 (IQR: −0.7 to 9.1) years after the first financing. Most drugs had no time between phase 1 and phase 2 given than 8 drugs either lacked a phase 1 trial or had combined phase 1/2 trials. There was a median of 4.2 (IQR: 3.5–4.9) years between the start of phase 3 trials and FDA approval.

**Table 4.** Time between development milestones and amount and proportion of investment received during each period (*n* = 9 companies).

	Years between each milestone,^a^ median (IQR)	Capital raised during period per company, millions		Percentage of total capital raised during period per company	
		Median (IQR)	Mean (SD)	Median (IQR)	Mean (SD)
Time between incremental development milestones					
First financing date to phase 1 start	5.1 (−0.7 to 9.1)	$107.9 ($47.0-$164.8)	$123.9 ($117.3)	17.0 (0.0-23.8)	23.7 (32.3)
Phase 1 start to phase 2 start	0.0 (0.0-0.7)	$0 ($0-$0)	$0 ($0)	0.0 (0.0-0.0)	0.0 (0.0)
Phase 2 start to phase 3 start	1.7 (1.2-3.2)	$245.9 ($53.0-$370.8)	$278.8 ($295.2)	31.8 (7.1-45.2)	30.4 (26.9)
Phase 3 start to FDA approval	4.2 (3.5-4.9)	$173.4 ($46.6-$702.3)	$696.7 ($1251.1)	34.7 (5.1-61.8)	39.4 (38.2)
Time between key milestones and FDA approval					
First financing date to FDA approval	13.5 (5.7-15.2)	$594.8 ($289.9-$908.7)	$1047.0 ($1332.2)	100.0 (100.0-100.0)	100.0 (0)
Phase 1 start to FDA approval	7.9 (4.8-10.7)	$356.2 ($230.9-$985.8)	$940.3 ($1325.9)	82.9 (76.1-94.0)	76.2 (32.3)
Granting of priority review to FDA approval	0.4 (0.4-0.7)	$0 ($0-$0)	$6.9 ($20.7)	0.0 (0.0-0.0)	0.7 (2.2)

Source: FDA drug approval databases, PitchBook investment deal data (investment amounts), and ClinicalTrials.gov (development milestone dates).

^a^Milestone dates correspond to each company's most recently approved index biologic.

Abbreviation: FDA, Food and Drug Administration.

Companies received a median of 17.0% (IQR: 0.0%–23.8%) of the total investment between the first financing transaction and phase 1, corresponding to $107.9 million (IQR: $47.0–$164.8 million), on average, per company. In contrast, 0% of the investment occurred between phase 1 and 2, given that most companies combined them. Between phase 2 and phase 3, companies received 31.8% (IQR: 7.1%–45.2%) of the total investment, a median of $245.9 million (IQR: $53.0–$370.8 million). Between phase 3 and FDA approval, 34.7% (IQR: 5.1%–61.8%) of the total investment occurred, a median of $173.4 million (IQR: $46.6–$702.3 million). Overall, companies received a median of 82.9% (IQR: 76.1%–94.0%) of their total investment, or $356.2 million (IQR: $230.9–$985.8 million), during the clinical trial and regulatory review stages of development from the start of phase 1 to FDA approval.

## Discussion

Despite policy interest in the cost of pharmaceutical innovation, little is known regarding the capital investment needed to bring a new innovative biologic drug to market among startups. We combined publicly available and proprietary data to estimate R&D costs for innovative biologic products developed by biotechnology startups. We estimated a median uncapitalized R&D investment of $304 million per biologic with priority review, or $873.7 million accounting for the cost of capital. The median time from phase 1 to FDA approval among these drugs was 7.9 years. In contrast with most prior studies, which relied on reported R&D expenditures in SEC filings, which are only available for public companies, our analysis introduces visibility into R&D investments among biotechnology startups during the period in which they are privately held through use of financial transaction data. This approach can inform ongoing policy efforts to estimate the cost of drug development and provide incentives for continued innovation. These results may also be a helpful benchmark for investors, startups, and drug discovery scientists.

Our sample selection criteria focusing on drugs approved through priority review produced a sample of drugs that all had orphan designation and other regulatory designations. Having orphan designation can help companies streamline the R&D process and reduce R&D costs.^31^ Small firms tend to focus on high-growth areas, including orphan drugs, which accounted for half of new active substances entering the US market in 2018.^10^

While there are many and varied prior estimates of drug development costs,^13-17^ ours are quantitatively closest to 2 studies. Prasad and colleagues^16^ estimated a median cost of $648 million in uncapitalized costs and $793 million in capitalized costs for new oncology drugs developed by companies with no prior FDA-approved drugs. These oncology drugs also all had orphan designation, suggesting that the similarities between the samples likely explain the similarities in the estimates. Second, an analysis by Sertkaya and colleagues^17^ of drugs across 13 therapeutic areas using clinical trial contracts containing per-patient R&D costs estimated development costs at $516 million, including the cost of failures but excluding the cost of capital. This estimate's similarity to our uncapitalized cost estimates supports our use of uncapitalized investments as a proxy for direct R&D costs. The capitalized cost estimate from that study, $879 million, was also close to our capitalized estimate of $873 million.

We focused exclusively on novel biologics, which are sometimes thought to be more difficult or expensive to develop than small molecules.^32^ Our estimate of the length of time between the start of phase 1 testing and FDA approval was similar to prior estimates across a range of drugs, and consistent with other studies indicating that, in fact, biologics that obtain FDA approval do not take more time to develop than small molecules.^8,17,33^ This suggests that incentives provided by the Orphan Drug Act, Bayh-Dole Act, SBIR, and R&D tax credits may be facilitating faster biologic development when the clinical value and public health implications of the biologic product are promising. These findings warrant future research and policy considerations about whether non-orphan innovative biologics are more resource intensive or more difficult for startups to develop, and if so, what policy incentives could facilitate biotechnology startup innovation in those disease areas.

There was variation between companies in the amount, type, and timing of investment. Several factors may account for the variation in R&D costs between companies in our sample, including variation in clinical trial costs,^34^ as well as the number of indications sought. For example, the startup with the highest investment per drug (argenx, $2.1 billion) currently has ongoing clinical trials of efgartimod alfa, their index biologic, for 11 additional indications.^35^ R&D investments are also a function of the ability to raise capital at a given point in time, which is affected by market conditions, experience of the management team, and the competitive landscape of a given therapeutic area.^36^

Of the total capital raised by these 9 companies, over 80% was raised during the clinical and regulatory stages of development, which is consistent with recent trends: VC investors are increasingly initiating funding during clinical rather than preclinical stages of development.^36^ It may also be more attractive to investors to shift investment timelines later, as the likelihood of technical success in phase 3 and subsequent FDA approval is nearly 60%, on average, across all drug disease areas.^17,37^ This also likely reflects that, for most of the startups, the drugs in the sample represented the startup's first FDA-approved drug. The period observed in our study thus represents the earlier years of company history.

Our estimates may inform policy efforts to improve incentives for pharmaceutical innovation. These results may inform efforts to quantify the effect of the Inflation Reduction Act on pharmaceutical innovation. Based on 1 study's estimates,^14^ the CBO estimated that 2 fewer drugs will be developed in the next decade, with greater decreases in the number of new drugs in subsequent decades.^38^ In revising this estimate, the CBO now intends to incorporate new estimates of the costs of drug development among publicly traded companies.^13,39^ The CBO could leverage our study's results to represent the cost of drug development among startups developing innovative biologics.

The results from our sample shed light on a component of the expanding landscape of pharmaceutical innovation, which consists of various models of drug development and financing that are shaped by policy incentives. While current policy discussions around biopharmaceutical innovation heavily focus on the R&D activities of large pharmaceutical manufacturers, policymakers should recognize that the market for drug innovation is increasingly complex, with various stakeholders. Both small and large companies play important and increasingly specialized roles in building the biopharmaceutical pipeline.^10,40^ Accordingly, the size and types of incentives needed may vary depending on the entities involved.

Our calculations may underestimate or overestimate the true costs of developing a novel FDA-approved biologic in different ways. Underestimation is possible because 3 of the products we examined—elivaldogene autotemcel, voretigene neparvovec, and tagraxofusp—originated at academic institutions and were sold or licensed to a startup during early development; our analyses do not capture R&D costs prior to start-up acquisition. In addition, we were unable to account for outside funding that 2 of the firms we examined received for 2 of the drugs: idecabtagene vicleucel was codeveloped by bluebird bio and Celgene and burosumab was codeveloped by Ultragenyx and Kyowa Kirin. On the other hand, overestimation is possible if some portion of R&D investments captured were used for new drug candidates that have not yet succeeded or failed.

Our study has other limitations. First, our data from Pitchbook did not allow us to disaggregate R&D investments across the drug candidates in a given company's portfolio; therefore, we calculated an average investment amount per drug by company. Second, we did not account for failures among companies that never bring a drug to market. While our cost estimates for successful biologics are lower than those reported in prior literature focusing on marketed drugs, our findings are not generalizable to biologics that failed to reach the market. Future research is needed to examine biologic development failures and compare them with those of small-molecule drugs. Third, our study period overlaps the COVID-19 pandemic, which may have caused costs and time required for development to increase for some drugs. Fourth, since we restricted our analysis to startups that retained ownership of a drug candidate throughout the entire development period to enable use of comparable company investment data, our sample size was necessarily restricted. The small sample size of mostly orphan drugs was also a limitation in other studies estimating the costs of R&D for high-cost cell and gene therapies and oncology drugs.^15,16^ Due to the sample, our estimates do not represent development costs for products developed by multiple companies through licensing partnerships, nor for products developed in-house or acquired by larger pharmaceutical companies. However, these results provide a first step in understanding the company financing required to develop innovative FDA-approved biologics among VC-backed biotechnology startups, and could pave the way for future analyses using these data to examine biotechnology startup companies that eventually become acquired or license their products.

## Conclusion

The median investment in a biotechnology startup needed to independently develop and commercialize an innovative FDA-approved biologic product was $304.1 million, accounting for failures in uncapitalized costs (representing the cost of R&D from the company perspective) and $873.7 million in capitalized costs (representing the investor perspective). The median time from the start of clinical trials to FDA approval was 7.9 years. A median of 82.9% of the total investment occurred during the clinical trial phase of development. These results provide new visibility into R&D investment needed among biotechnology startups and may help inform the decisions of investors, startups, and policymakers interested in promoting pharmaceutical innovation.

## Supplementary Material

qxaf139_Supplementary_Data

## Data Availability

The raw Pitchbook data is proprietary however our tables and eFigures provide information on development milestone dates, investment amounts, and types of investments.
