# Supplementary material for: Research and development investments for biologics independently developed by US biotechnology startups, 2017–2023
Source: Health Aff Sch. 2025 Jul 25;3(7):qxaf139. doi: 10.1093/haschl/qxaf139 (PMC12290397; doi:10.1093/haschl/qxaf139)
Supplement: qxaf139_Supplementary_Data [file qxaf139_supplementary_data.zip › online_supplement_biotech_drug_dev_05-15-2025.docx]

**Research and Development INVESTMENTS For BiologicS INDEPENDENTLY developed by U.S. Biotechnology Startups, 2017-2023**

**Online Supplement**

eFigure 1. Sample selection.

13 index biologics from 9 companies that had a record of venture capital backing in PitchBook data

13 index biologics from 9 companies that were privately held at the start of development of the index biologic and retained ownership of the index biologic from development to approval

91 index biologics from 67 companies (the group identified in previous step excluding discontinued, vaccine, and tissue products)

103 biologics with priority review FDA-approved between 2017-2023 from 74 companies

eFigure 2. Investment amount over time by investment type.

**
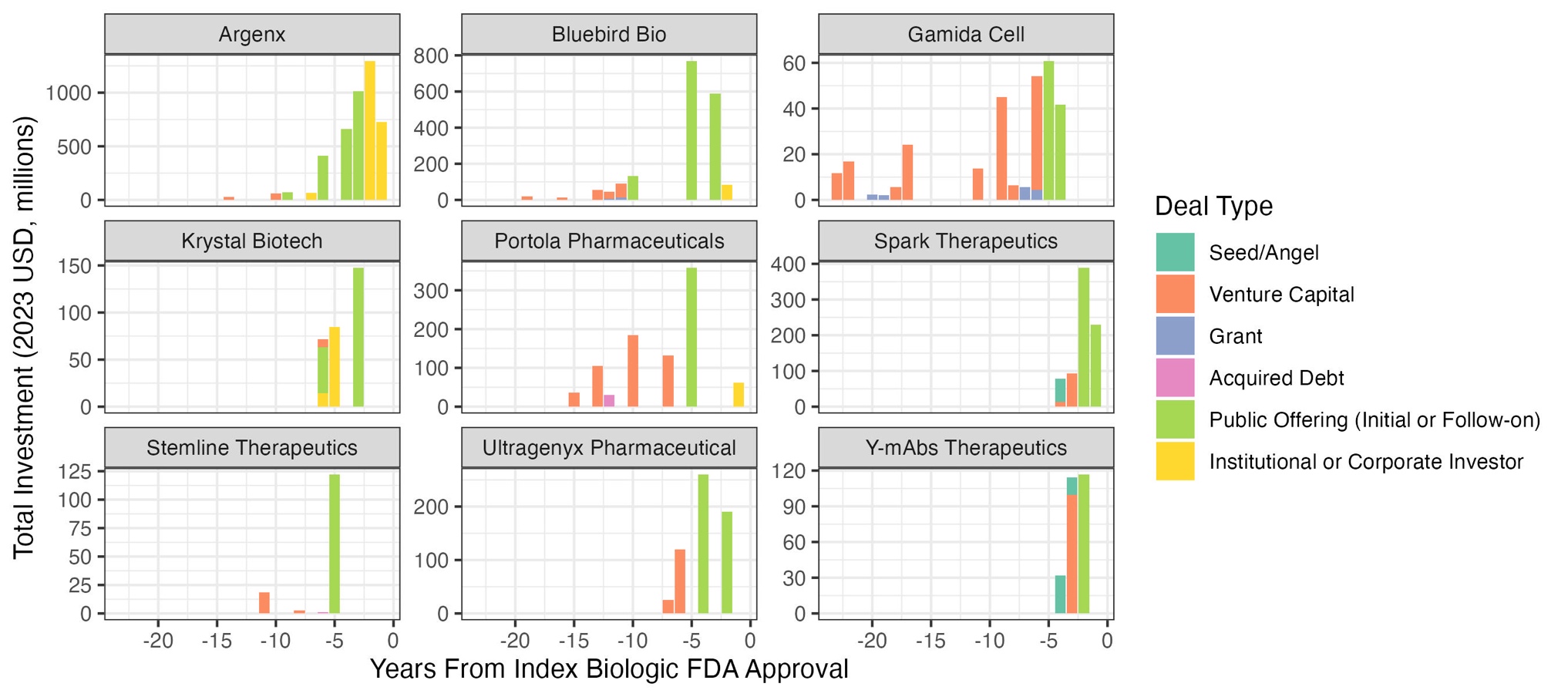
**

Source: PitchBook® investment deal data

Year 0 represents the year of the most recent index biologic FDA approval
